# Heme Oxygenase-1 Regulates Dendritic Cell Function through Modulation of p38 MAPK-CREB/ATF1 Signaling*

**DOI:** 10.1074/jbc.M113.532069

**Published:** 2014-04-09

**Authors:** Laith M. A. Al-Huseini, Han Xian Aw Yeang, Junnat M. Hamdam, Swaminathan Sethu, Naif Alhumeed, Wai Wong, Jean G. Sathish

**Affiliations:** From the ‡ Medical Research Council (MRC) Centre for Drug Safety Science and Department of Molecular and Clinical Pharmacology, Sherrington Buildings, Ashton Street, University of Liverpool, Liverpool L69 3GE, United Kingdom and; the § Department of Pharmacology and Therapeutics, College of Medicine, Al-Qadisiyah University, P. O. Box 80, Diwaniyah 58001, Iraq

**Keywords:** CREB, Dendritic Cells, Heme Oxygenase, p38 MAPK, Reactive Oxygen Species (ROS), ATF1, Co-stimulation

## Abstract

Dendritic cells (DCs) are critical for the initiation of immune responses including activation of CD8 T cells. Intracellular reactive oxygen species (ROS) levels influence DC maturation and function. Intracellular heme, a product of catabolism of heme-containing metalloproteins, is a key inducer of ROS. Intracellular heme levels are regulated by heme oxygenase-1 (HO-1), which catalyzes the degradation of heme. Heme oxygenase-1 has been implicated in regulating DC maturation; however, its role in other DC functions is unclear. Furthermore, the signaling pathways modulated by HO-1 in DCs are unknown. In this study, we demonstrate that inhibition of HO-1 activity in murine bone marrow-derived immature DCs (iDCs) resulted in DCs with raised intracellular ROS levels, a mature phenotype, impaired phagocytic and endocytic function, and increased capacity to stimulate antigen-specific CD8 T cells. Interestingly, our results reveal that the increased ROS levels following HO-1 inhibition did not underlie the changes in phenotype and functions observed in these iDCs. Importantly, we show that the p38 mitogen-activated protein kinase (p38 MAPK), cAMP-responsive element binding protein (CREB), and activating transcription factor 1 (ATF1) pathway is involved in the mediation of the phenotypic and functional changes arising from HO-1 inhibition. Furthermore, up-regulation of HO-1 activity rendered iDCs refractory to lipopolysaccharide-induced activation of p38 MAPK-CREB/ATF1 pathway and DC maturation. Finally, we demonstrate that treatment of iDC with the HO-1 substrate, heme, recapitulates the effects that result from HO-1 inhibition. Based on these results, we conclude that HO-1 regulates DC maturation and function by modulating the p38 MAPK-CREB/ATF1 signaling axis.

## Teaser

**Background:** HO-1 contributes to redox homeostasis and regulation of immature dendritic cell (DC) phenotype.

**Results:** HO-1 inhibition results in increased ROS, activation of p38 MAPK-CREB/ATF1 pathway, and dysregulation of DC phenotype and function.

**Conclusion:** HO-1 influences DC function through effects on p38 MAPK-CREB/ATF1 signaling pathway.

**Significance:** This study provides new insights into the molecular pathways influenced by HO-1 in DCs.

## Introduction

Dendritic cells (DCs)^4^ are potent antigen-presenting cells that play a major role in the initiation and regulation of the immune response (1). Immature DCs (iDCs) are efficient at capturing extracellular antigens through several endocytic and phagocytic mechanisms (2, 3). However, iDCs are poorly immunogenic as they express low levels of MHC class II molecules and co-stimulatory receptors including CD86 and CD40 at the cell surface. Dendritic cell maturation is triggered through engagement of pattern recognition receptors such as the Toll-like receptors (TLRs) by pathogen-associated molecular patterns, *e.g.* bacterial lipopolysaccharide (LPS). Maturation is associated with morphological, phenotypic, and functional changes including up-regulation of cell surface MHC II, co-stimulatory molecules, loss of phagocytic capacity, and enhanced antigen-presenting capabilities, which are required for inducing competent T cell activation (4–6). A key intracellular signaling pathway that governs DC maturation is the p38 MAPK pathway (7). The p38 MAPK signaling pathway positively regulates DC phenotype and cytokine production by driving the expression of multiple genes involved in DC maturation (8). The major transcription factors that lie downstream of the p38 MAPK pathway are the cAMP-response element-binding protein (CREB) and activation transcription factor 1 (ATF1) (9, 10). Dendritic cell maturation and function are influenced by changes in cellular levels of reactive oxygen species (ROS) (11). Alterations in intracellular ROS can also impact on the activity of the p38 MAPK pathway (12). Heme is a product of catabolism of heme-containing metalloproteins that has the potential to induce the generation of ROS (13). Accumulation of intracellular heme is prevented by the enzymatic activity of heme oxygenases (HO), in particular, the HO-1 isoform (14). Heme oxygenase-1 catalyzes the degradation of heme into biliverdin (BV), free iron, and carbon monoxide (CO) (15). We and others have identified a potential role for HO-1 in regulating DC maturation (16, 17). However, it is unclear whether HO-1 utilizes the p38 MAPK pathway to mediate regulation of DC maturation. Furthermore, it is not known whether the primary effect of HO-1 in regulating DC maturation is by preventing elevation of intracellular ROS levels. Finally, it is unknown whether the HO-1 substrate, heme, is directly involved in regulating DC maturation and function through activation of the p38 MAPK-CREB/ATF1 pathway. In this study, we show that inhibition of HO-1 activity in mouse bone marrow-derived iDCs results in a mature phenotype associated with an impaired phagocytic and endocytic capacity and an enhanced ability to stimulate antigen-specific T cell proliferation. We also demonstrate that although HO-1 inhibition was accompanied by elevated ROS, the increased ROS was not required for inducing DC maturation. Importantly, we show that the DC maturation and functional changes brought about by HO-1 inhibition are mediated through the p38 MAPK-CREB/ATF1 pathway. Finally, we provide evidence for the induction of p38 MAPK-CREB/ATF1 activation and DC maturation by the HO-1 substrate, heme. We conclude that HO-1 regulates DC maturation and function by modulating the p38 MAPK-CREB/ATF1 signaling axis.

## EXPERIMENTAL PROCEDURES

### 

#### 

##### Reagents

All reagents were from Sigma-Aldrich unless otherwise stated. FCS, Dextran^FITC^ (40,000 *M*_r_), and carboxyfluorescein succinimidyl ester (CFSE) (Invitrogen); tin protoporphyrin IX dichloride (SnPP-IX) and cobalt(III) protoporphyrin IX chloride (CoPP) (Tocris Bioscience, Bristol, UK); and SB203580 (Cell Signaling Technology, Danvers, MA) were also purchased for the study.

##### Mice

Mice transgenic for the H-2D^b^-restricted T cell receptor (TCR)-αβ transgene, F5, were a kind gift from Dr. James Matthews (Cardiff, Wales, UK). Mice were maintained at the Biomedical Services Unit, University of Liverpool. Protocols described herein were undertaken in accordance with criteria outlined in the license granted under the Animals (Scientific Procedures) Act 1986 (PPL 40/3379).

##### Generation of Bone Marrow-derived DCs

Mouse bone marrow-derived iDCs were generated according to published protocol (18).

##### Cell Surface Receptor Expression

DCs were stained with fluorescent αCD11c^TC^ (Invitrogen) and αCD86^FITC^ or αMHC II^PE^ (BD Biosciences) antibodies for 30 min on ice, washed, acquired on a BD FACSCanto II flow cytometer (BD Biosciences), and analyzed using Cyflogic software (version 1.2.1 CyFlo Ltd.).

##### DC Phagocytosis Assay

Apoptotic thymocytes were generated from mouse thymi treated with 1 μm dexamethasone for 18 h. Apoptotic thymocytes were labeled with the intracellular fluorescent dye, CFSE, and co-cultured with plate-adherent DCs for 2 h at 37 or 4 °C. Cells were stained with αCD11c^TC^ prior to analysis by flow cytometry. Jurkat T cells were fluorescently labeled with CFSE, and necrosis was induced by snap-freezing in liquid nitrogen. Necrotic cells were co-cultured with DCs as above and analyzed by flow cytometry.

##### DC Endocytosis Assay

DCs were incubated with 0.5 μg/ml Dextran^FITC^ (40,000 *M*_r_) for different time points at 37 °C. Cells were stained for surface expression of CD11c as described above and analyzed by flow cytometry.

##### Measurement of ROS

Untreated or treated iDCs were stained using the fluorescent ROS indicator, dihydroethidium, according to Ref. 19 and analyzed by flow cytometry.

##### F5 CD8 T Cell Proliferation

F5 CD8 T cell proliferation was quantified as described previously (20). Briefly, iDCs were pulsed with a concentration range of antigenic peptide (NP68), washed, and co-cultured with F5 CD8 T cells for 72 h. [^3^H]Thymidine was added for the last 16 h. Cells were harvested onto glass fiber filter mats and read on a scintillation counter (MicroBeta TriLux; PerkinElmer Life Sciences, Buckinghamshire, UK).

##### Gel Electrophoresis and Western Immunoblotting

DCs were lysed, and 5 μg of lysate protein was resolved by SDS-PAGE, transferred to PVDF membranes (Bio-Rad; Hertfordshire, UK), blocked, and probed for proteins of interest using the appropriate primary antibodies (phospho-p38 MAPK and phospho-CREB (Cell Signaling Technology, Danvers, MA) and α-tubulin (Santa Cruz Biotechnologies)) followed by horseradish peroxidase-conjugated secondary antibodies (Cell Signaling Technology) and visualized using the ECL system (PerkinElmer Life Sciences).

##### Statistics

Raw data obtained were analyzed using unpaired *t* test, one-way ANOVA and the Mann-Whitney *U* test. *p* values < 0.05 were considered to be statistically significant.

## RESULTS

### 

#### 

##### HO-1 Inhibition Alters Immature DC Phenotype and Function

**FIGURE 1.**
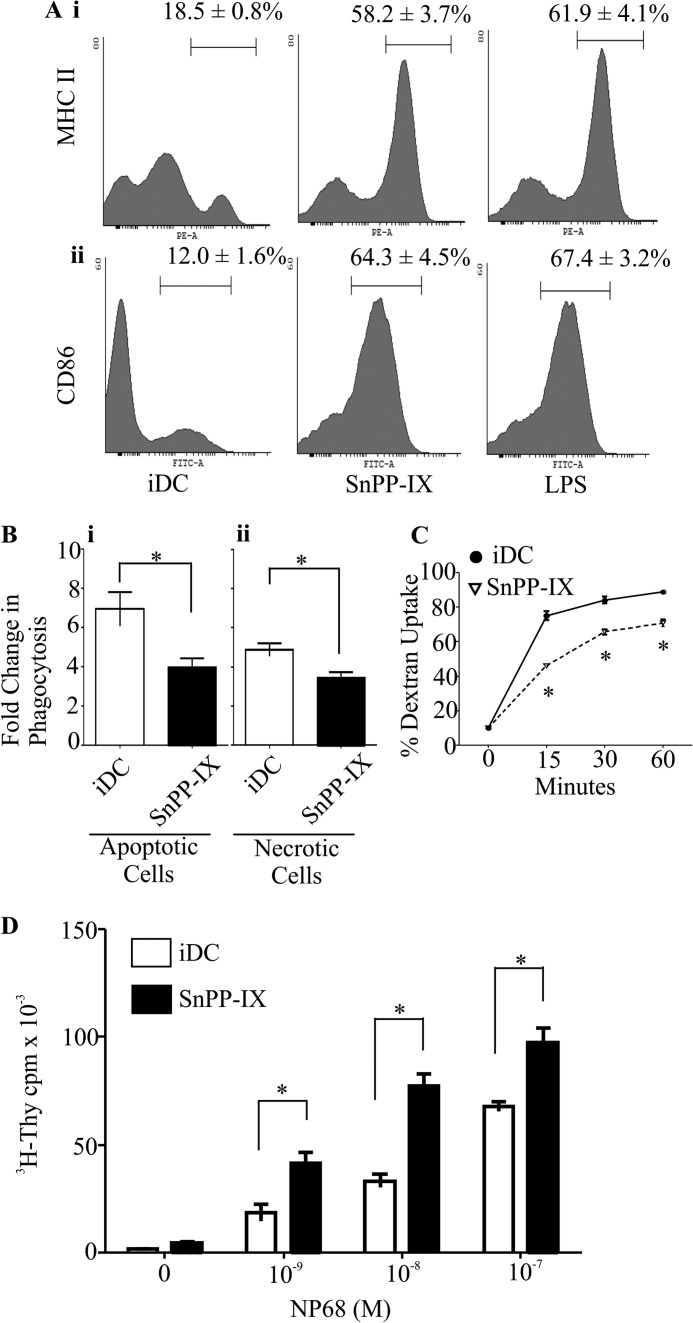
**Inhibiting HO-1 activity alters dendritic cell phenotype and function.** *A*, immature DCs untreated or treated with HO-1 inhibitor (SnPP-IX, 5 μm) or LPS (1 μg/ml) for 14 h. Cells were labeled with fluorescent conjugated antibodies against MHC II and CD86 co-stimulatory molecules. Co-stimulatory molecule expression was determined by flow cytometry. The percentages of iDCs expressing high levels of MHC II (*panel i*) and CD86 (*panel ii*) are indicated above the marker. Representative histograms are presented with average percentage ± S.D. Data are derived from three independent experiments. *B,* immature DCs untreated or treated with SnPP-IX (5 μm) for 14 h were co-cultured with CFSE-labeled necrotic Jurkat cells (*panel i*) or apoptotic thymocytes (*panel ii*) at 37 °C for 2 h. DC phagocytic capacity was measured by flow cytometry as an increase in CFSE levels when compared with corresponding 4 °C baseline control samples. Data derived from four independent experiments are presented as average -fold changes ± S.E. Statistical significance was tested by Mann-Whitney *U* test (*, *p* < 0.05). *C*, endocytic capacity was measured by incubating iDCs untreated or treated with SnPP-IX (5 μm) for 14 h with Dextran^FITC^ for the indicated time points at 37 °C. Dextran^FITC^ uptake by iDCs was assessed by flow cytometry. Data derived from three independent experiments are presented as average percentage of uptake ± S.D. Statistical significance was tested by unpaired Student's *t* test (*, *p* < 0.05). *D*, immature DCs untreated or treated with SnPP-IX (5 μm) for 14 h were pulsed with increasing concentrations of NP68 antigenic peptide and co-cultured with F5 CD8 T cells for 72 h. [^3^H]Thymidine (*^3^H-Thy*) was added for the last 16 h. Proliferation of T cells was determined by scintillation counting of incorporated [^3^H]thymidine. Data are presented as average scintillation counts ± S.D. Statistical significance was assessed using one-way ANOVA. Data are representative of three independent experiments (*, *p* < 0.05).

To investigate the role of HO-1 in DC maturation and function, we treated iDCs with the inhibitor of HO-1 activity, SnPP-IX. Inhibition of HO-1 by SnPP-IX in iDCs resulted in increased levels of MHC II and CD86 cell surface expression in comparison with untreated DCs (Fig. 1*A, panel i*, MHC II 58.2 ± 3.7% *versus* 18.5 ± 0.8%, *p* < 0.05; Fig. 1*A, panel ii*, CD86 64.3 ± 4.5% *versus* 12.0 ± 1.6%, *p* < 0.05). Furthermore, MHC II and CD86 levels on SnPP-IX-treated iDCs were comparable with LPS-treated iDCs (MHC II 61.9 ± 4.1% and CD86 67.4 ± 3.2%). Following maturation, DCs have reduced phagocytic and endocytic capabilities (21, 22). As HO-1 inhibition resulted in DC maturation, we examined the antigen acquisition capacity of SnPP-IX-treated iDCs. Our results revealed that SnPP-IX-treated iDCs had a reduced capacity to phagocytose both necrotic cells (Fig. 1*B, panel i,* 4.9 ± 0.7 *versus* 3.4 ± 0.6-fold increase over baseline, *p* < 0.05) and apoptotic cells (Fig. 1*B, panel ii,* 6.9 ± 1.7 *versus* 4.0 ± 0.5-fold increase over baseline, *p* < 0.05) when compared with their untreated control. Results also demonstrated that SnPP-IX-treated iDCs had a diminished capacity to endocytose dextran in comparison with their untreated controls (Fig. 1*C*, 46.0 ± 1.0% *versus* 75.0 ± 2.6% at 15 min, 65.7 ± 1.5% *versus* 84.0 ± 2.0% at 30 min, and 70.7 ± 2.1% *versus* 88.7 ± 1.2% at 60 min, *p* < 0.05). Low levels of co-stimulatory molecule expression in iDCs render them unable to stimulate a fully competent antigen-specific CD8 T cell response (23). However, up-regulation of these surface molecules in mature DCs enhances their ability to induce T cell activation (24). As SnPP-IX-treated iDCs exhibit a mature phenotype, we expected that this would be associated with an enhanced capacity to induce DC-mediated antigen-specific CD8 T cell activation. To test this, we utilized a TCR transgenic mouse model, F5, wherein the CD8 T cells exclusively express the F5 T cell receptor (F5 TCR) that specifically recognizes the MHC-I (H2-D^b^)-restricted antigenic peptide, NP68, when presented by DCs (25). Functional consequences of altered DC co-stimulatory receptor expression were assessed by the ability of NP68-bearing DCs to stimulate antigen-specific F5 CD8 T cell proliferation. We observed that SnPP-IX-treated DCs elicited enhanced DC-mediated antigen-specific F5 CD8 T cell proliferation in relation to the untreated control at all NP68 concentrations as shown in Fig. 1*D* (2.5-fold at 1 and 10 nm to 1.4-fold at 100 nm, *p* < 0.05). Taken together, these findings indicate that inhibition of HO-1 activity affects DC phenotypic maturation, antigen acquisition ability, and antigen-specific CD8 T cell stimulatory capacity.

##### HO-1 Inhibition in DCs Results in Elevated Intracellular ROS Levels

**FIGURE 2.**
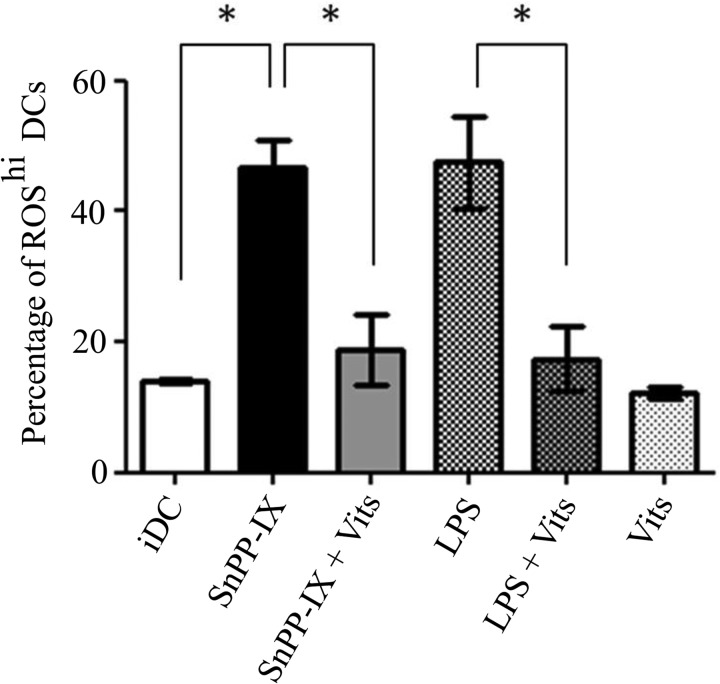
**Inhibiting HO-1 activity increases DC intracellular ROS levels that can be reversed by antioxidant vitamins.** Immature DCs were untreated or treated with SnPP-IX (5 μm) for 14 h or in combination with vitamins (*Vits*) C (1 mm) and E (100 μm) for 48 h, with LPS (1 μg/ml) alone for 2 h or in combination with vitamins C and E. DCs were incubated with the fluorescent ROS indicator, dihydroethidium, and analyzed by flow cytometry. Data derived from three independent experiments are presented as the percentage of cells with high ROS (*ROS^hi^*) levels ± S.D. (*, *p* < 0.05).

Dendritic cell maturation and function are influenced by intracellular ROS levels (26). To test whether HO-1 activity is required for regulation of ROS levels in DCs, we treated iDCs with SnPP-IX and measured the ROS levels using the fluorescent redox-sensitive probe, dihydroethidium. Dihydroethidium reports on superoxide levels, and superoxide is a key ROS that has been shown to induce the maturation of DCs (26). We found that HO-1 inhibition resulted in a significant increase in intracellular ROS levels in iDCs as shown in Fig. 2 (46.6 ± 8.7% *versus* 14.0 ± 0.7%, *p* < 0.05), an increase similar to that observed in the LPS-treated iDCs (47.6 ± 14.2%). Intracellular ROS can be lowered by ROS scavengers such as vitamins C and E (16), and here we observed that treatment with vitamins C and E resulted in significant reduction in ROS levels induced by SnPP-IX treatment in iDCs (Fig. 2, 18.8 ± 9.4% *versus* 46.6 ± 8.7%, *p* < 0.05). Similar effects were also observed in LPS- and vitamin-treated iDCs (Fig. 2). These findings implicate HO-1 activity in preventing elevation of intracellular ROS levels in iDCs.

##### Altered Immature DC Function by HO-1 Inhibition Is Not Dependent on Elevated ROS

**FIGURE 3.**
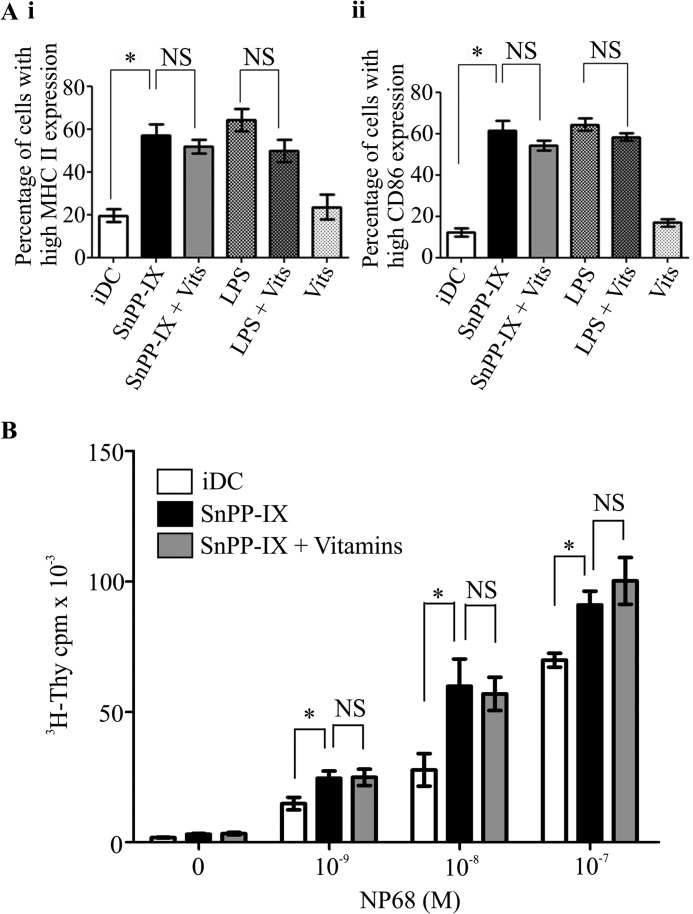
**Altered immature DC function by HO-1 inhibition is not dependent on elevated ROS.** *A*, immature DCs were untreated or treated with SnPP-IX (5 μm) or LPS (1 μg/ml) for 14 h alone or in combination with vitamins (*Vits*) C (1 mm) and E (100 μm) for 48 h plus SnPP-IX (5 μm) for 14 h. MHC II (*panel i*) and CD86 (*panel ii*) expression was determined by flow cytometry and presented as the percentage of cells expressing high MHC II or CD86. Data derived from three independent experiments are presented as average percentage ± S.D. (*, *p* < 0.05; *NS*, not significant). *B*, immature DCs untreated or treated with SnPP-IX (5 μm) for 14 h alone or treated with vitamins C (1 mm) and E (100 μm) for 48 h along with SnPP-IX (5 μm) for the last 14 h. Cells were pulsed with increasing concentrations of NP68 antigenic peptide and co-cultured with F5 CD8 T cells for 72 h. [^3^H]Thymidine (*^3^H-Thy*) was added for the last 16 h. Proliferation of T cells was determined by scintillation counting of incorporated [^3^H]thymidine. Data are presented as average scintillation counts ± S.D. Statistical significance was assessed using one-way ANOVA. Data are representative of three independent experiments (*, *p* < 0.05; *NS*, not significant).

To test whether DC maturation induced by HO-1 inhibition was a result of increased intracellular ROS levels, we treated HO-1-inhibited iDCs with vitamins C and E and examined MHC II and CD86 expression. Our results revealed that there were no significant differences between SnPP-IX treatment alone or in combination with vitamins C and E in iDC expression of MHC II (Fig. 3*A, panel i,* 57.2 ± 9.0% *versus* 51.8 ± 5.6%, *p* > 0.05) and CD86 (Fig. 3*A, panel ii,* 61.6 ± 9.1% *versus* 54.2 ± 4.9%, *p* > 0.05). In addition, no significant differences in the expression of MHC II and CD86 was observed in iDCs treated either with LPS alone or with LPS and vitamins C and E (Fig. 3*A, panel i,* 64.2 ± 9.0% *versus* 49.9 ± 9.1%, *p* > 0.05 and Fig. 3*A, panel ii,* 64.4 ± 6.0% *versus* 58.3 ± 3.6%, *p* > 0.05, respectively). Similarly, there were no differences observed in iDC-mediated antigen-specific F5 CD8 T cell proliferation upon SnPP-IX treatment alone or in combination with vitamins C and E (Fig. 3*B*). These observations suggest that the altered iDC phenotype and function induced by HO-1 inhibition are not a result of elevated ROS in these iDCs.

##### HO-1 Regulates DC Phenotype and Function through the p38 MAPK-CREB/ATF1 Pathway

**FIGURE 4.**
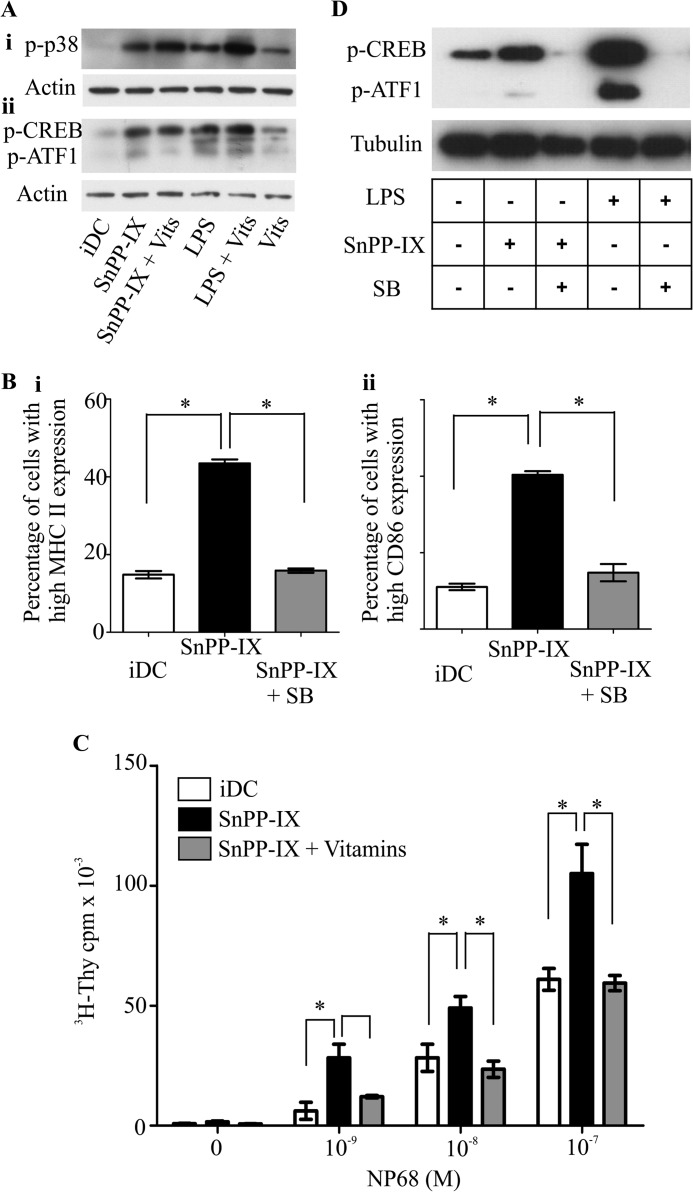
**Modulation of dendritic cell phenotype and function by HO-1 is mediated through the p38 MAPK-CREB/ATF1 pathway.** *A*, cell lysates generated from iDCs that were pretreated or not with vitamins (*Vits*) C and E (48 h) and then left untreated or treated with SnPP-IX (5 μm) for 2 h or with LPS (1 μg/ml) for 30 min were subjected to SDS-PAGE, and levels of phosphorylated p38 MAPK (*p-p38*) (*panel i*) and phosphorylated CREB (*p-CREB*) (*panel ii*) followed by actin were assessed by Western blotting. *p-ATF1*, phosphorylated ATF1. *B*, immature DCs untreated or treated with SnPP-IX (5 μm) for 14 h alone or treated with p38 MAPK activity inhibitor SB203580 (*SB*, 20 μm) for 48 h along with SnPP-IX (5 μm) for the last 14 h. MHC II (*panel i*) and CD86 (*panel ii*) expression was determined by flow cytometry and presented as the percentage of cells expressing high MHC II or CD86. Data derived from three independent experiments are presented as average percentage ± S.D. (*, *p* < 0.05). *C*, immature DCs were untreated or treated with SnPP-IX (5 μm) for 14 h or treated with SB203580 (20 μm) for 48 h along with SnPP-IX (5 μm) for the last 14 h. DCs were then pulsed with increasing concentrations of NP68 antigenic peptide and co-cultured with F5 CD8 T cells for 72 h. [^3^H]Thymidine (*^3^H-Thy*) was added for the last 16 h. Proliferation of T cells was determined by scintillation counting of incorporated [^3^H]thymidine. Data are presented as average scintillation counts ± S.D. Statistical significance was assessed using one-way ANOVA. Data are representative of three independent experiments (*, *p* < 0.05). *D*, cell lysates were generated from iDCs untreated or treated with SnPP-IX (5 μm) for 2 h in the presence or absence of SB203580 (20 μm) for 1 h prior to SnPP-IX treatment. These iDCs were subjected to SDS-PAGE, and phosphorylation status of CREB and ATF1 (*p-CREB* and *p-ATF1*) was assessed by Western blotting. Lysates from iDCs treated with SB203580 (20 μm) for 1 h in the presence or absence of LPS (1 μg/ml) for the last 30 min were also included. Tubulin was assessed for equal loading of lanes.

Activation of the p38 MAPK-CREB/ATF1 signaling pathway has been shown to be involved in DC maturation (27). Activation of this pathway is accompanied by an increase in the serine phosphorylation status of p38 MAPK (28, 29). To investigate whether p38 MAPK is involved in the HO-1-mediated regulation of DC function, we first examined the phosphorylation status of p38 MAPK upon SnPP-IX treatment. As demonstrated in Fig. 4*A, panel i,* HO-1 inhibition resulted in a marked increase in p38 MAPK phosphorylation, and this was independent of ROS status, as we found that the increased p38 MAPK phosphorylation following HO-1 inhibition remained unaffected when iDCs were co-treated with vitamins and SnPP-IX (Fig. 4*A, panel i*). We then examined the phosphorylation status of proteins that are downstream of p38 MAPK, *i.e.* CREB/ATF-1 (Fig. 4*A, panel ii*). We observed a marked induction in phospho-CREB/ATF-1in iDCs treated with SnPP-IX, and no difference was noted in this phosphorylation status when the cells were treated with vitamins along with SnPP-IX. A similar pattern was observed with LPS stimulation. We then assessed the requirement of the p38 MAPK pathway for the induction of DC maturation elicited by HO-1 inhibition using a pharmacological inhibitor of p38 MAPK (SB203580) and examined its effects on SnPP-IX-treated iDC phenotype and function. As shown in Fig. 4*B*, inhibition of p38 MAPK activity prevented the increase in co-stimulatory molecule expression induced by SnPP-IX treatment of iDCs (MHC II 43.5 ± 1.0 *versus* 15.9 ± 0.6%, *p* < 0.05, *panel i*; CD86 40.4 ± 0.9% *versus* 14.8 ± 2.3%, *p* < 0.05, *panel ii*). Furthermore, p38 MAPK inhibition also prevented the enhanced antigen-specific F5 CD8 T cell proliferation mediated by SnPP-IX-treated iDCs (Fig. 4*C*, reduction of 2.5-fold at 1 nm NP68, *p* < 0.05, 2-fold at 10 nm NP68, *p* < 0.05, and 1.4-fold at 100 nm, *p* < 0.05, which are comparable with iDCs). To test whether inhibition of p38 MAPK activity in SnPP-IX-treated DCs, which resulted in reduced DC maturation phenotype, is manifested through altered CREB/ATF1 signaling, DCs were treated with SnPP-IX and SB203580. Western immunoblotting revealed that SnPP-IX treatment resulted in increased CREB/ATF1 phosphorylation (Fig. 4*D*). Furthermore, the enhanced CREB/ATF1 phosphorylation was markedly reduced by p38 MAPK inhibition. Collectively, these results suggest that HO-1 regulates DC phenotype and function through modulation of the p38 MAPK-CREB/ATF1 signaling axis.

##### Antioxidant N-Acetylcysteine (NAC) Does Not Reverse Effects of HO-1 Inhibition in Immature DCs

**FIGURE 5.**
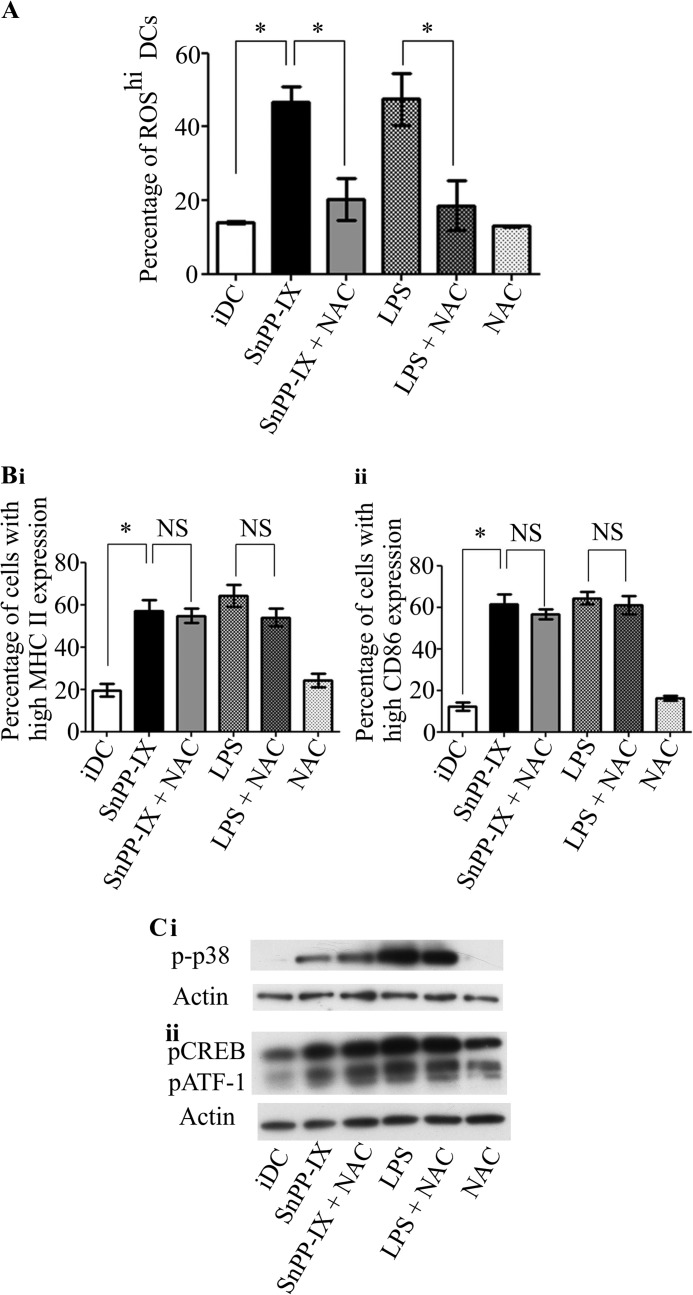
**Antioxidant NAC does not reverse the effects of HO-1 inhibition in immature DCs.** *A*, immature DCs were untreated or treated with SnPP-IX (5 μm) for 14 h or in combination with NAC (25 μm, 48 h) or with LPS alone (1 μg/ml) for 2 h or in combination with NAC (25 μm, 48 h). DCs were incubated with the fluorescent ROS indicator, dihydroethidium, and analyzed by flow cytometry. Data derived from three independent experiments are presented as the percentage of cells with high ROS levels ± S.D. (*, *p* < 0.05). *B*, immature DCs were untreated or treated with SnPP-IX (5 μm) or LPS (1 μg/ml) for 14 h alone or in combination with NAC (25 μm, 48 h). MHC II (*panel i*) and CD86 (*panel ii*) expression was determined by flow cytometry and presented as the percentage of cells expressing high MHC II or CD86. Data derived from three independent experiments are presented as average percentage ± S.D. (*, *p* < 0.05; *NS*, not significant). *C*, cell lysates generated from iDCs that were pretreated or not with NAC (25 μm, 48 h) and then left untreated or treated with SnPP-IX (5 μm) for 2 h or with LPS (1 μg/ml) for 30 min were subjected to SDS-PAGE, and levels of phosphorylated p38 MAPK (*p-p38*) (*panel i*) and phosphorylated CREB (*pCREB*) and actin (*panel ii*) were assessed by Western blotting. *pATF1*, phosphorylated ATF1.

To test whether the effects of HO-1 inhibition in DCs can be reversed by antioxidants other than vitamins, we used NAC as an additional ROS scavenger. NAC has been shown to be a potent ROS scavenger in DCs (30) that can elevate or replenish intracellular glutathione levels (31). We observed that, as with vitamins C and E, NAC was effective in lowering the increased intracellular ROS levels induced by SnPP-IX in iDCs (Fig. 5*A*, *SnPP-IX versus SnPP-IX* + *NAC* 46.6 ± 8.7% *versus* 20.2 ± 9.8%, *p* < 0.05). To further confirm the effects that ROS reduction by NAC have on DC maturation, we treated iDCs with NAC alone and in combination with SnPP-IX. Our results showed that there were no significant differences between the expression of MHC II and CD86 in SnPP-IX-treated iDCs or SnPP-IX- and NAC-treated iDCs (Fig. 5*B, panel i*, 57.2 ± 9.0% *versus* 54.7 ± 5.8%, *p* > 0.05 and Fig. 5*B, panel ii*, 61.6 ± 9.1% *versus* 56.7 ± 4.7%, *p* > 0.05 respectively). We next examined whether NAC affects the SnPP-IX-mediated activation of p38 MAPK-CREB/ATF1 pathway in DCs. Results showed that NAC does not affect the phosphorylation of p38 MAPK and CREB/ATF1 induced by SnPP-IX (Fig. 5*C*). NAC had similar effects on LPS-induced changes in DC phenotype and signaling. These results further support the proposition that the effects of HO-1 inhibition on DCs are not dependent on ROS.

##### Up-regulation of HO-1 Renders DCs Refractory to LPS-induced DC Signaling and Maturation

**FIGURE 6.**
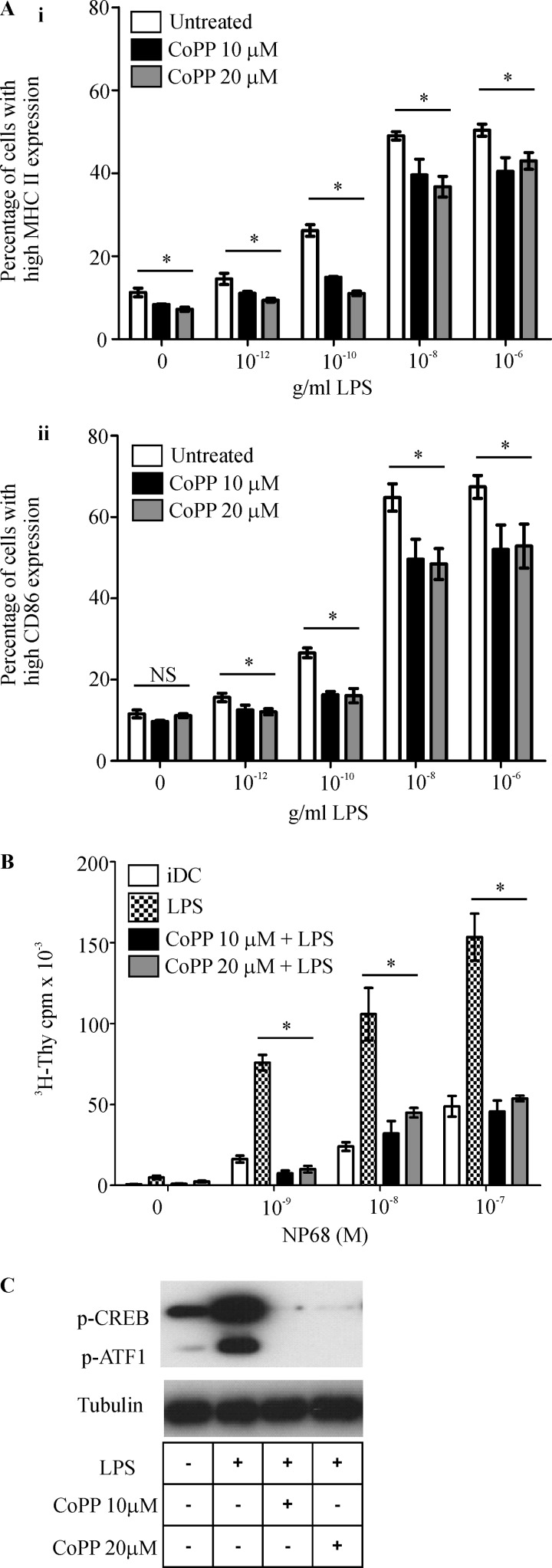
**Up-regulation of HO-1 activity inhibits LPS-induced signaling, phenotypic, and functional changes in DCs.** *A*, immature DCs untreated or treated with HO-1 inducer CoPP (10 and 20 μm) for 24 h with or without LPS (1 μg/ml) for 18 h at the indicated concentrations. MHC II (*panel i*) and CD86 (*panel ii*) expression was determined by flow cytometry and presented as the percentage of cells expressing high MHC II or CD86. Data derived from three independent experiments are presented as average percentage ± S.D. (*, *p* < 0.05; *NS*, not significant). *B*, immature DCs were untreated or treated with CoPP (10 and 20 μm) for 24 h along with or without LPS (1 μg/ml) for the last 18 h. DCs were then pulsed with increasing concentrations of NP68 antigenic peptide and co-cultured with F5 CD8 T cells for 72 h. [^3^H]Thymidine (*^3^H-Thy*) was added for the last 16 h. Proliferation of T cells was determined by scintillation counting of incorporated [^3^H]thymidine. Data are presented as average scintillation counts ± S.D. Statistical significance was assessed using one-way ANOVA. Data are representative of three independent experiments (*, *p* < 0.05). *C*, cell lysates generated from iDCs untreated or treated with CoPP (10 and 20 μm) for 4 h in combination with LPS (1 μg/ml) for 30 min. Lysates were subjected to SDS-PAGE, and phosphorylation status of CREB and ATF1 (*p-CREB* and *p-ATF1*) was assessed by Western blotting. Lysate from iDCs treated with LPS (1 μg/ml) for 30 min was used as positive control. Tubulin was assessed for equal loading of lanes.

To further test the involvement of HO-1 in the regulation of DC phenotype and function through p38 MAPK-CREB/ATF1, we investigated the effect of HO-1 up-regulation on LPS-triggered DC maturation and CREB/ATF1 phosphorylation. CoPP can be used to up-regulate the expression of HO-1 (32). We demonstrated that both basal and LPS-induced up-regulation of MHC II molecules expression was significantly reduced (Fig. 6*A, panel i*) when iDCs were treated with CoPP (at concentrations of 10 and 20 μm). HO-1 induction also causes significant reduction in LPS-induced up-regulation of CD86 (Fig. 6*A, panel ii*). Furthermore, CoPP treatment resulted in a significant reduction in LPS-treated DC-mediated antigen-specific F5 CD8 T cell proliferation (Fig. 6*B*.) To evaluate the influence of CoPP treatment on LPS-induced phosphorylation of CREB and ATF1, iDCs were treated with CoPP and stimulated with LPS. Cobalt protoporphyrin treatment markedly reduced CREB/ATF1 phosphorylation in LPS-treated DCs as shown in Fig. 6*C*. These results strengthen the evidence for the regulation of DC function by HO-1.

##### The HO-1 Substrate, Heme, Induces DC Functional Maturation through p38 MAPK-CREB/ATF1 Pathway

**FIGURE 7.**
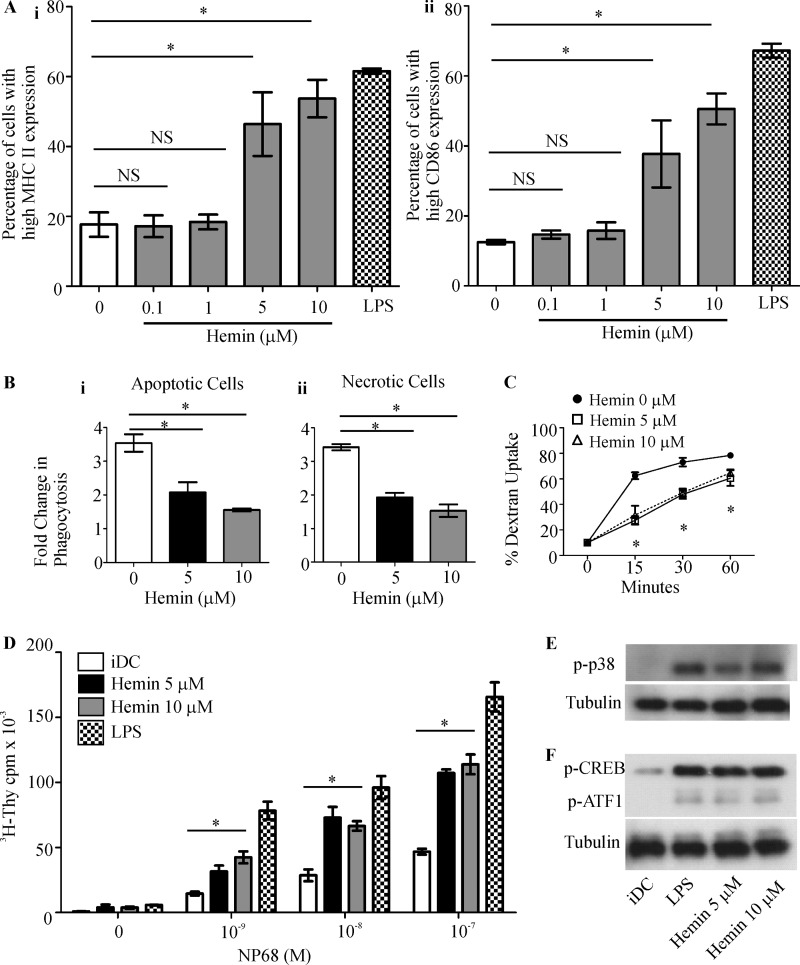
**HO-1 substrate, heme, induces iDC signaling and phenotypic and functional changes in DCs.** *A*, immature DCs untreated or treated with hemin (0.1, 1, 5, and 10 μm) or LPS (1 μg/ml) for 14 h. MHC II (*panel i*) and CD86 (*panel ii*) expression was determined by flow cytometry and presented as the percentage of cells expressing high MHC II or CD86. Data derived from three independent experiments are presented as average percentage ± S.D. (*, *p* < 0.05; *NS*, not significant). *B*, immature DCs untreated or treated with hemin (5 and 10 μm) for 14 h were co-cultured with CFSE-labeled necrotic Jurkat cells (*panel i*) or apoptotic thymocytes (*panel ii*) at 37 °C for 2 h. DC phagocytic capacity was measured by flow cytometry as an increase in CFSE levels when compared with corresponding 4 °C baseline control samples. Data derived from four independent experiments are presented as average -fold changes ± S.E. Statistical significance was tested by Mann-Whitney *U* test (*, *p* < 0.05). *C*, endocytic capacity was measured by incubating iDCs with Dextran^FITC^ for the indicated time points at 37 °C. Dextran^FITC^ uptake by iDCs was assessed by flow cytometry. Data derived from three independent experiments are presented as average percentage of uptake ± S.D. Statistical significance was tested by unpaired Student's *t* test (*, *p* < 0.05). *D*, immature DCs were untreated or treated with hemin (5 and 10 μm) or with LPS (1 μg/ml) for 14 h. DCs were then pulsed with increasing concentrations of NP68 antigenic peptide and co-cultured with F5 CD8 T cells for 72 h. [^3^H]Thymidine (*^3^H-Thy*) was added for the last 16 h. Proliferation of T cells was determined by scintillation counting of incorporated [^3^H]thymidine. Data are presented as average scintillation counts ± S.D. Statistical significance was assessed using one-way ANOVA. Data are representative of three independent experiments (*, *p* < 0.05). *E*, cell lysates were generated from iDCs untreated or treated with hemin (5 and 10 μm) or LPS (1 μg/ml) for 30 min. Lysates were subjected to SDS-PAGE, and phosphorylation status of p38 MAPK (*p-p38*) was assessed by Western blotting. Tubulin was assessed for equal loading of lanes. *F*, cell lysates were generated from iDCs untreated or treated with (5 and 10 μm) hemin or LPS (1 μg/ml) for 30 min and subjected to SDS-PAGE, and phosphorylation status of CREB and ATF1 (*p-CREB* and *p-ATF1*) was assessed by Western blotting. Tubulin was assessed for equal loading of lanes.

Inhibition of HO-1 is thought to result in intracellular accumulation of the HO-1 substrate, heme (33). When heme (as hemin, the oxidized form of heme), is added to innate immune cells, it can accumulate within the cells (34–36). We therefore used hemin to test whether it can recapitulate the effects of HO-1 inhibition on DC phenotype and function. When iDCs were treated with hemin, the expression of MHC II and CD86 was up-regulated when compared with untreated iDCs (MHC II 17.7 ± 3.5% (untreated) *versus* 46.4 ± 9.1% (at 5 μm) and 53.7 ± 5.4% (at 10 μm), *p* < 0.05, Fig. 7*A, panel i*; CD86, 12.5 ± 0.6% (untreated) *versus* 37.7 ± 9.6% (at 5 μm) and 50.6 ± 4.4% (at 10 μm), *p* < 0.05, *panel ii*). The mature phenotype observed in hemin-treated DCs was comparable with that of LPS-treated DCs (MHC II 61.6 ± 0.7%, and CD86 67.2 ± 2.0%). Next we investigated the effect of hemin on DC phago- and endocytic function. Our results revealed that DCs treated with hemin exhibited a reduction in their ability to phagocytose necrotic cells (1.93 ± 0.31 for 5 μm hemin and 1.53 ± 0.41 for 10 μm hemin when compared with 3.42 ± 0.21-fold increase over the baseline for untreated iDCs, *p* < 0.05, Fig. 7*B, panel i*) and apoptotic cells (2.07 ± 0.61 for 5 μm hemin and 1.55 ± 0.09 for 10 μm hemin when compared with 3.54 ± 0.52-fold increase over the baseline for untreated iDCs, *p* < 0.05, Fig. 7*B, panel ii*). A similar reduction was observed in the endocytic capacity of hemin-treated iDCs (Fig. 7*C*, 27.5 ± 3.3% for 5 μm hemin and 31.5 ± 7.4% for 10 μm hemin *versus* 62.5 ± 2.7% for iDCs at 15 min, 47.9 ± 3.5% for 5 μm hemin and 49.2 ± 3.2% for 10 μm hemin *versus* 73.1 ± 3.4% for iDCs at 30 min, and 60.6 ± 6.1% for 5 μm hemin and 64.7 ± 2.7% for 10 μm hemin *versus* 78.4 ± 1.6% for iDCs at 60 min, *p* < 0.05). Furthermore, hemin-treated iDCs demonstrated an enhanced capacity to stimulate antigen-specific F5 CD8 T cell proliferation when compared with untreated iDCs (Fig. 7*D).* Finally, we investigated the effects of hemin on the p38 MAPK pathway in iDCs. We demonstrated that p38 MAPK (Fig. 7*E*), CREB, and ATF1 (Fig. 7*F*) were hyperphosphorylated in hemin-treated DCs. Taken together, these results suggest that the changes in DC phenotype, function, and signaling induced by HO-1 inhibition could be attributable to the effects of the HO-1 substrate, heme.

## DISCUSSION

HO-1 is an important stress-inducible enzyme that mediates antioxidant and cytoprotective effects resulting in maintenance of cellular redox homeostasis and protects cells from oxidative stress (37, 38). In this study, we have shown the contribution of HO-1 in the regulation of DC phenotype and immune function. Furthermore, we provide evidence for the involvement of the p38 MAPK-CREB/ATF1 signaling axis in mediating the effects of HO-1 on DC function. Inhibition of HO-1 activity in iDCs was found to cause an enhanced maturation phenotype in terms of increased levels of MHC II and CD86 expression consistent with previous studies (16, 17, 39, 40). To further delineate the precise role of HO-1 in iDC immune function, we examined the impact of HO-1 inhibition on antigen acquisition and iDC-mediated antigen-specific CD8 T cell stimulatory capacity. HO-1 inhibition resulted in impaired phagocytic and endocytic function in iDCs. This is most likely to be a consequence of the enhanced maturation status of these DCs rather than direct regulation by HO-1 of these biological processes.

Effector cytotoxic T lymphocyte generation, from naive CD8 T cells, requires strong co-stimulatory signals in addition to TCR signals provided by mature DCs (41–43). Consequently, the mature phenotype exhibited by the SnPP-IX-treated DC was associated with increased CD8 T cell stimulation capacity. In addition to increased co-stimulation, it is possible that HO-1-inhibited DCs may secrete proinflammatory cytokines that would further influence the quality and magnitude of the CD8 T cell response. Cytokine analysis of the supernatants from HO-1-inhibited iDCs should provide data on this aspect. Immature DCs, which have low levels of co-stimulatory molecule expression, contribute to immune tolerance by engaging with self-reactive CD8 T cells and causing CD8 T cell anergy or deletion (44). This implicates HO-1, through its effect on DC co-stimulatory molecule expression, in the maintenance of immune tolerance mediated by iDCs. Interestingly, there is evidence for the requirement of HO-1 function for the expansion of a CD4 subset of immunosuppressive T cells, regulatory T cells (Tregs), the generation of which is dependent on iDCs (40).

The induction of HO-1 is dependent on the activity of the transcription factor, Nrf2 (45). We have previously shown that iDCs, which lack Nrf2, display an enhanced maturation phenotype and increased T cell stimulatory capacity that is remarkably similar to DCs treated with SnPP-IX (46). This suggests that lowered HO-1 activity due to loss of Nrf2 underlies the phenotypic and functional changes observed in the Nrf2-deficient iDCs. The mechanism by which HO-1 activity contributes to the maintenance of DCs in an immature state merits investigation. The enzymatic action of HO-1 on its substrate heme leads to generation of CO and BV (15). One possible mechanism through which HO-1 regulates DC function is through generation of CO and BV. We hypothesize that upon inhibition of HO-1 activity in iDCs, deprivation of HO-1 products CO and BV in iDCs leads to enhanced maturation and T cell stimulatory function of the DCs. In support of this, previous studies have shown that CO inhibited DC maturation through TLR3 and TLR4 pathways (47) and BV administration protected against LPS-induced shock (48). In keeping with these findings, we have also demonstrated that induction of HO-1 in DCs using CoPP was associated with lower sensitivity to LPS-induced DC maturation. The potential increase in CO production as a result of higher HO-1 activity in iDCs treated with CoPP may underlie the resistance to TLR4 signaling by LPS. This could be brought about through the interaction of CO with caveolin-1 (cav-1), a structural component of the plasma membrane that when bound to TLR4 in the presence of CO results in inhibition of LPS-induced signaling (49). Another potential mechanism for the effects of HO-1 on DC maturation could be through its role in redox homeostasis. Changes in ROS levels have been implicated in many aspects of DC biology including cell maturation and cytokine production (26). We demonstrated that HO-1 inhibition increased ROS levels in iDCs possibly due to buildup of intracellular heme. Heme can directly induce intracellular ROS generation (50). However, lowering ROS levels through vitamin and NAC treatment did not prevent DC maturation induced by HO-1 inhibition, indicating that these changes in DC phenotype and function are not ROS-mediated. Consistent with the lack of ROS involvement in mediating these changes in iDCs, similar findings were observed in our previous study, which demonstrated that resetting elevated ROS levels in Nrf2-deficient DCs did not reverse the dysregulated DC phenotype and function (16). Although in our study, we measured superoxide ROS, our findings can extend to other ROS as vitamins (C and E) and NAC have been demonstrated to scavenge a large variety of ROS (30, 51).

The molecular pathways that are utilized by HO-1 to modulate DC function were a focus of our investigations. Studies that used pharmacological inhibition of p38 MAPK have demonstrated the importance of this pathway in up-regulation of DC co-stimulatory molecules induced by LPS (8). In the current study, p38 MAPK inhibition prevented the mature DC phenotype in SnPP-IX-treated DCs. Furthermore, p38 MAPK inhibition also produced a marked reversal in the enhanced DC-mediated antigen-specific CD8 T cell proliferation observed in SnPP-IX-treated iDCs. In addition, the downstream effectors of p38 MAPK, CREB and ATF1, were also activated in SnPP-IX-treated iDCs, as revealed by their hyperphosphorylated state, which could be reduced by the p38 MAPK inhibitor. These results highlight the role for p38 MAPK-CREB/ATF1 activity in mediating the effects of HO-1 on DC function. The exact molecular basis for p38 MAPK-CREB/ATF1 activation in HO-1-inhibited DCs is not known, but one possibility could be due to interaction of intracellular heme with receptors that signal through this pathway. We tested this possibility by treating DCs with heme and demonstrated that not only did heme activate the p38 MAPK-CREB/ATF1 pathway but it also recapitulated the other phenotypic and functional effects of HO-1 inhibition in DCs (enhanced co-stimulatory receptor expression, enhanced CD8 T cell stimulatory capacity, and reduced endo- and phagocytic capability). Heme has been shown to engage and activate TLR4, thereby activating signaling pathways including the p38 MAPK-CREB/ATF1 axis (17). We therefore speculate that elevated level of heme in HO-1-inhibited DCs activates TLR4. This could be tested by examining whether the altered phenotype, function, and signaling seen in HO-1-inhibited DCs are abolished when HO-1 is inhibited in TLR4-deficient DCs.

HO-1 is being considered as a viable therapeutic target for a variety of conditions or disorders involving the immune system, including organ transplantation and inflammatory disorders (52), and therefore it is critical to understand the details of HO-1-mediated regulation of DC function. Our observations provide further details and insight into the role of HO-1 in DC biology and could therefore inform the design of pharmacological strategies that aim to modulate DC function in the therapy of immune diseases.

## Footnotes

4 The abbreviations used are:

DC: dendritic cell
iDC: immature dendritic cell
HO-1: heme oxygenase-1
Nrf2: nuclear factor-erythroid 2 (NF-E2) p45-related factor-2
ROS: reactive oxygen species
NP68: nucleoprotein 68
NAC: *N*-acetylcysteine
CREB: cAMP-response element-binding protein
ATF1: activating transcription factor 1
BV: biliverdin
CFSE: carboxyfluorescein succinimidyl ester
TLR: Toll-like receptor
TCR: T cell receptor
CoPP: cobalt(III) protoporphyrin IX chloride
SnPP-IX: tin protoporphyrin IX dichloride
ANOVA: analysis of variance.
